# Physeal-Sparing Rigid Intramedullary Nailing in Adolescent Tibial Shaft Fractures: A Pilot Study

**DOI:** 10.7759/cureus.13893

**Published:** 2021-03-15

**Authors:** Kevin A Williams, Zachary T Thier, Candler G Mathews, Mark D Locke

**Affiliations:** 1 Orthopaedic Surgery, University of Alabama at Birmingham School of Medicine, Birmingham, USA; 2 Medical Education, Lincoln Memorial University DeBusk College of Osteopathic Medicine, Knoxville, USA; 3 Orthopaedic Surgery, University of Arkansas for Medical Sciences, Little Rock, USA; 4 Orthopaedic Surgery, Prisma Health - University of South Carolina, Columbia, USA

**Keywords:** adolescent tibial fracture, trauma pediatric, intramedullary nailing

## Abstract

Purpose: Elastic stable intramedullary nailing (ESIN) and open reduction internal fixation (ORIF) are literature-supported operative treatments for displaced tibial shaft fractures in skeletally immature patients. Very little is written about rigid intramedullary nails (RIMNs) in adolescents. Our purpose is to describe a physeal-sparing, reamed, locked RIMN technique for adolescent tibial shaft fractures and report its safety.

Methods: Adolescent patients with tibial shaft fractures indicated for operative intervention at one institution were retrospectively identified from 2011-2018. Patients were classified based on method of fracture fixation. Primary outcomes included fracture union, reoperation, and complication rates.

Results: Thirteen patients were included in the RIMN arm, with an average age of 13.8 years. Two patients in the observational group underwent ESIN and seven patients underwent ORIF, with an average age of 11.5 years. Significant differences were found between time of immobilization (28 days vs 121 days), time to touch down weight bearing release (1 day vs 34 days), and hardware pain (2/13 vs 7/9). The RIMN group sustained fewer reoperations (2/13 vs 5/9). No differences were found in rates of complications or fixation failure between groups.

Conclusions: Based on our small pilot study, RIMNs in adolescents should be considered as a potential treatment option when a physeal-sparing distal start point is utilized. Additionally, short-term follow-up suggests safety. Patients who underwent the RIMN procedure required fewer reoperations compared with the observational group. Overall, fracture healing was similar across the two groups. The benefits of RIMN include early immobilization and improved weight-bearing profile.

Level of Evidence: IV.

## Introduction

This article was previously presented as a meeting abstract at the 2019 South Carolina Orthopedic Association Annual Meeting in August 2019 and at the 2020 Southern Orthopaedic Association Annual Meeting in July 2020. Tibial shaft fractures are among the most common pediatric fractures, making up approximately 15% of long bone fractures in this population. They are both the third most common pediatric fracture in general and the third most common fracture in a multiply injured pediatric patient [1]. The mainstay of treatment for closed tibial shaft fractures remains closed reduction and cast application. While less than 5% of these fractures require surgery, certain indications necessitate surgery including open injuries, irreducible fractures, polytrauma, and floating knees, amongst others. Unstable tibial shaft fractures have historically been treated with elastic stable intramedullary nailing (ESIN), external fixation, or open reduction internal fixation (ORIF) with plates and screws. Complications associated with these include pin site infection, nonunion, overgrowth, and refracture [2-4].

ESIN and ORIF are both literature-supported operative treatments for displaced tibial shaft fractures in adolescents with open physes [5]. Multiple studies have shown that ESIN has a faster time to union than casting or external fixation [2,6-8]. A study on ESIN demonstrated that the time needed to progress to full weight-bearing was 8.4 weeks on average and all patients had their elastic nails removed, on average, 23.1 weeks after the initial surgery, with two patients losing reduction and required subsequent manipulation under anesthesia [4]. Additional benefits of ESIN include better alignment, relatively quicker weight-bearing, and improved range of motion compared with casting or external fixation [2,9,10]. While some studies have shown problems with loss of reduction using ESIN for femur fractures in older and heavier patients, there are no studies showing loss of reduction in this demographic for ESIN in the tibia [9-15].

Hardware irritation remains one of the most common complications in most studies evaluating the usage of ESIN. Often, this requires hardware removal. Most techniques describe leaving approximately two cm of nail protrusion to minimize irritation and allow for future hardware removal, a procedure that occurs nearly ubiquitously within the pediatric orthopedic community and can be considered one of the disadvantages to using the ESIN technique [1-4]. Another disadvantage is the need for supplemental immobilization and delaying weight bearing. Most studies within the literature demonstrated the need for short leg splinting or casting with both ESIN and ORIF techniques [14,15].

In the adult population, rigid, locked intramedullary nailing exists as the gold standard for treating tibial shaft fractures [16]. Like ESIN, this technique allows for preservation of the fracture hematoma and a closed insertion compared to plate and screw fixation. Additionally, RIMN permits earlier weight bearing, adds more rotational stability, and may treat more proximal or distal fractures via blocking screws and other advanced nailing techniques [17,18]. Very little is written about rigid intramedullary nailing (RIMN) in adolescents, given the transphyseal nature of a traditional tibial nail starting point. This obviates the need for consideration of other techniques given the potential for subsequent physeal arrest with the transphyseal technique [19]. One group studied the use of RIMN in adolescents and did not show an increased incidence of growth arrest, but their technique was not physeal-sparing [20]. Our purpose is to describe a physeal-sparing reamed locked RIMN technique and report its safety in adolescent tibial shaft fractures. 

## Materials and methods

Following internal review board approval, a retrospective review was completed at a single level two pediatric trauma center. All adolescent patients with tibial shaft fractures requiring operative intervention at one institution by four different surgeons were retrospectively identified from 2011-2018. Patients were included if: operative fixation occurred within the timeline, there were at least eight weeks of follow-up data with radiographs, they had open growth plates, and the fractures did not involve either tibial growth plate.

**Figure 1:**
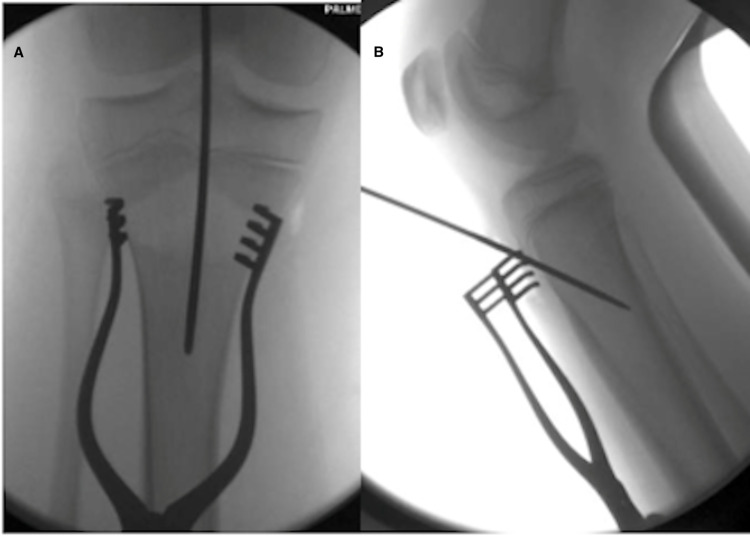
Antero-posterior (A) and lateral (B) fluoroscopic images of the proximal tibial starting point for RIMN RIMN: rigid intramedullary nailing

**Figure 2:**
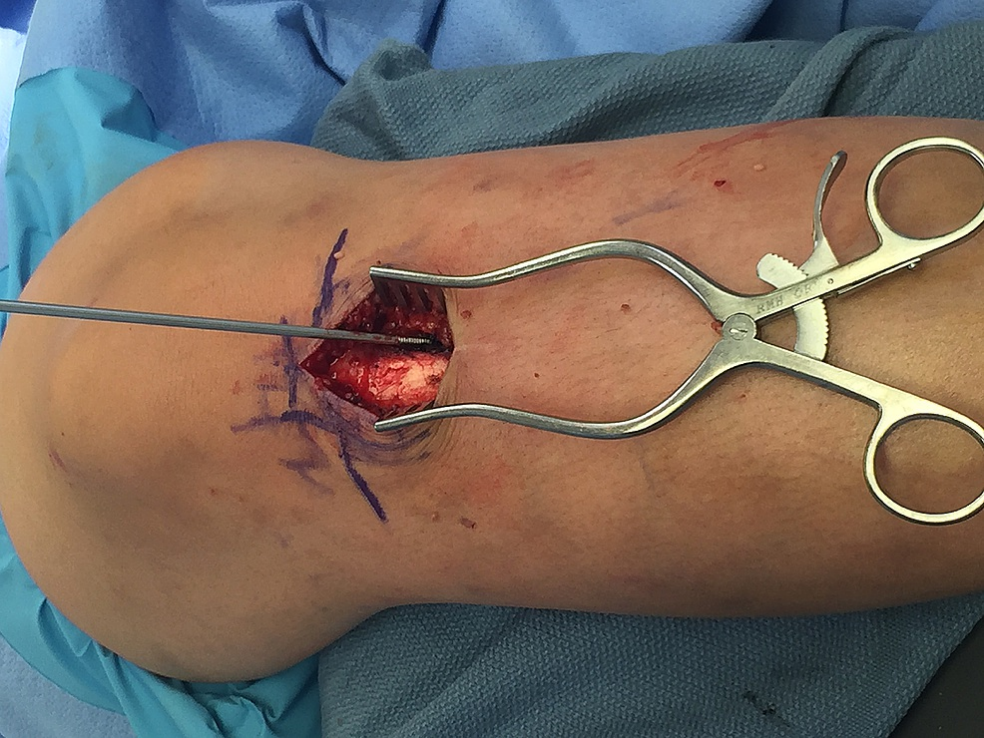
Tibial nail starting point, just lateral to the tibial tubercle apophysis and distal to the proximal tibial physis.

**Figure 3:**
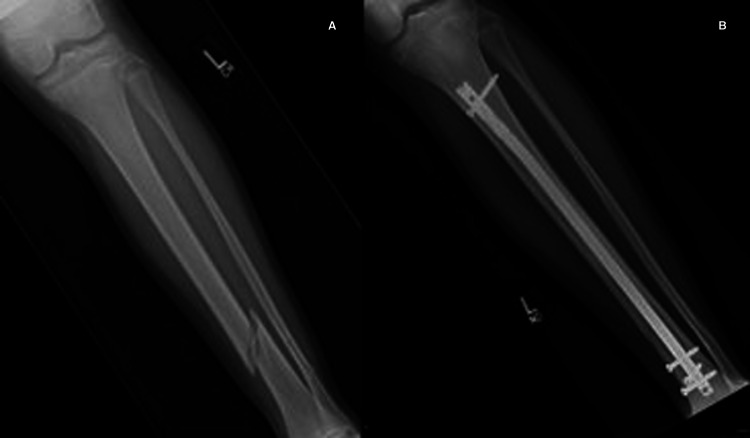
Pre- (A) and post-operative (B) images of physeal-sparing RIMN RIMN: rigid intramedullary nailing

Intramedullary nailing was performed via a physeal-sparing technique as developed by the senior author. Synthes tibial nails were used; mostly 8 mm in diameter based on pre-operative, calibrated measurements. The operative leg was flexed over a radiolucent triangle and several landmarks were established radiographically including the physis, the intramedullary axis of the tibia, and the tibial tubercle apophysis. An incision was made along the medial border of the tibial tubercle just distal to the proximal tibial physis. Full-thickness skin flaps were created, and the periosteum was elevated near the site of threaded-tip guidewire placement. The 3.2 mm guidewire (TZ1) was placed approximately three cm distal to the proximal tibial physis in line with the medullary axis of the tibia which lies medial to the tibial tubercle apophysis. The guidewire angle must be steep in order to protect the posterior tibial cortex. Guidewire placement must be confirmed fluoroscopically in both planes (Figures 1, 2). Depending on the proximity to the tibial tubercle, one may use the cannulated awl or the opening reamer. Once the opening was created, rigid tibial nailing and interlocking proceeded in the routine manner (Figure 3). We elected to ream to 1.5 mm above the intended nail size during RIMN implantation in accordance with findings from the Study to Prospectively Evaluate Reamed Intramedullary Nails in Patients with Tibial Fractures (SPRINT) trial [18].

Patients were classified based on fracture fixation. ORIF and ESIN patients were included in our observational group and RIMN patients comprised our experimental group for analysis. Their charts were evaluated to identify follow-up, mechanism of injury, background demographics (including age and sex), complications, need for reoperation, total length of immobilization (short leg walking cast and/or controlled ankle motion [CAM] walker boot), and release to touch down weight-bearing status following operative fixation. Radiographs were then reviewed for fracture union, radiographic complications, and presence of growth arrest.

MATLAB R2019a V.9.6 (MathWorks, Natick, MA, USA) was used for statistical analysis. One-sided Wilcoxon rank sum tests were performed to compare weight bearing and immobilization between groups. Fisher’s exact tests were utilized for analysis on the incidence of postoperative complications and eventual need for reoperation.

## Results

Thirteen patients were included in our experimental RIMN arm, all of them male ranging in ages 13-17 at time of surgery. The average age and BMI of the RIMN group were 13.8 years and 21.66, respectively. Two patients in the observational group underwent ESIN and seven patients underwent ORIF with plate and screws. Ages ranged from eight to 14 at time of surgery with an average age of 11.6 years. The observational group on average had a higher BMI at 24.36 vs 21.66 as demonstrated in Table 1.

**Table 1:** Demographic characteristics between groups RIMN: rigid intramedullary nailing; ESIN: elastic stable intramedullary nailing, ORIF: open reduction internal fixation

	RIMN (N=13)	ESIN/ORIF (N=9)
Age (yrs)	13.77 ± 1.3	11.56 ± 2.07
BMI	21.66 ± 3.03	24.36 ± 5.93
Sex (% male)	100	55.56

**Figure 4:**
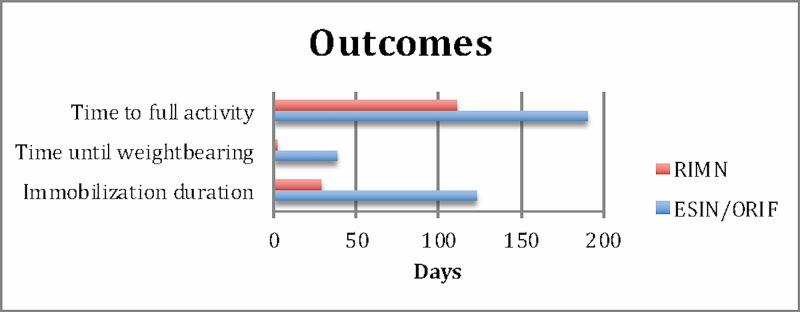
Selected outcomes between groups RIMN: rigid intramedullary nailing; ESIN: elastic stable intramedullary nailing, ORIF: open reduction internal fixation

Significant differences were found between time of immobilization (121 days vs 28 days; p <.05), time until touch down weight bearing release (34 days vs 1 day; p <.05), and hardware pain (78% vs 15%; p <.05) of the observational group and RIMN group respectively. The RIMN group demonstrated a decreased need for reoperation (15% vs 56%; p=.07). No differences were found in rates of complications, physeal arrest, or fixation failure between groups. (Figure 4, Table 2). Minimum follow-up was 11 weeks and nine weeks for RIMN and observational groups, respectively. Maximum follow-up was 160 and 348 weeks for RIMN and observational groups, respectively. Mean follow-up was 43.5 and 108.1 weeks for RIMN and observational groups, respectively.

**Table 2:** Findings between groups RIMN: rigid intramedullary nailing; ESIN: elastic stable intramedullary nailing, ORIF: open reduction internal fixation

	RIMN (N=13)	ESIN/ORIF (N=9)	P
Time of immobilization (days)	28.08 ± 31.46	122.75 ± 120.83	0.009
Time to weight bearing (days)	0.92 ± 2.56	38 ± 17.73	< 0.001
Time to full activity (days)	111.25 ± 52.08	190 ± 135.97	0.072
Fixation failure (%)	7.69	22.23	0.544
Hardware pain (%)	15.38	77.78	0.007
Reoperation (%)	15.38	55.56	0.074

## Discussion

Our pilot study aimed to initially answer that question. Additional studies within the last decade have illuminated the increase in operative fixation of tibial shaft fractures [21-23]. Our study initially suggests a safe, effective physeal-sparing distal starting point that can be utilized with minimal complications and the benefits that rigid, interlocked nailing can provide in the treatment of adolescent tibial shaft fractures.

In the adult population, rigid interlocked nailing has demonstrated multiple benefits over ORIF including preservation of the fracture hematoma, increased rotational stability, and earlier weight bearing. Its closed nature precipitates closed reduction techniques or percutaneous open reductions, decreasing the number of incisions and associated complication rates. Reamed nailing has been proven to be beneficial to union rates in closed tibial shaft fractures in adults, according to the SPRINT trial. Reaming does disrupt the endosteal blood supply, but it also provides a significant amount of growth factors to the fracture site. Additionally, reaming increases the canal diameter and therefore allows for placement of a larger diameter nail. Increased nail diameter results in larger diameter interlocking screws and subsequently more rotational stability. Theoretically, these advantages improve the bony union profile of the fracture to achieve a callous and thus secondary healing [17,18].

This was reviewed in more detail by Srivastava et al. who evaluated 24 patients undergoing ESIN. They demonstrated ESIN as an acceptable treatment option that provides some of the healing benefits of closed nailing, without the rigidity and stability provided by RIMN [24]. More recently, Pennock et al. provided a retrospective review of 44 patients undergoing ESIN and 26 undergoing ORIF. They showed no difference in healing rates but revealed a decreased immobilization period, angular deformity incidence, and reoperation rate within their ORIF group. They also found a higher rate of wound complications and operative times within the ORIF group. Their cast duration averaged 10.5 weeks for the ESIN group and seven weeks for the ORIF group. They report their weight bearing restriction time as 8.5 weeks for ESIN and 6.6 weeks for ORIF. Both data points were statistically significant [11].

In our cohort, several different types of immobilization were recorded, including CAM walker boots, splints, and casts. Despite our small sample size, we noted significant differences in time of immobilization between groups (121 days vs 28 days; p<.05). One patient in the observational group underwent hemiepiphysiodesis and thus was immobilized for 415 days, increasing the length of immobilization for the observational group moderately. Given the difference in immobilization and the more rigid fixation provided by RIMN, earlier weight bearing was encouraged within the experimental group. This resulted in earlier release to touch down weight bearing in this group compared with our observational (34 days vs 1 day; p<.05). We believe this represents an addition to the current literature regarding the operative treatment of tibial shaft fractures in adolescents.

The amount of hardware pain within the observational group seemed relatively high compared with rates in current literature (15% for RIMN group vs 78% for our observational group; p<.05). This likely contributed to the high rate of removal of hardware in this group. Overall, the reoperation rate was higher within the observational group, likely due to an increased number of hardware removals (15% in the RIMN group vs 56% in ESIN/ORIF group; p=.07). As demonstrated in multiple studies, many institutions plan for routine removal of hardware between six and 12 months postoperatively and this paradigm is reflected during placement of the hardware. Surgeons tend to leave elastic nails more superficial if they intend for future removal [4]. Additionally, given the more intramedullary and less superficial location of the hardware, the senior author does not routinely remove the rigid intramedullary nails. One patient in the RIMN group underwent rigid nail dynamization for a hypertrophic nonunion. One malunion was present after ORIF in the observational group and this patient went on to undergo medial distal hemiepiphysiodesis for ankle valgus. No additional differences were found in fixation failure or union rates between groups. Our population did not have any cases of compartment syndrome, which fails to echo the current literature rates of nearly 20%, but our sample size is quite small comparatively [25].

Several study limitations are present. First, our pilot study represents a small sample size from a relatively modest area, which could inhibit our ability to generalize the results. This must be kept in mind when evaluating our conclusions. Although our pilot study represents a low-powered patient sample size, several statistically significant results were obtained. Overall, our patient cohort mimicked the reported demographic in the current literature of a preponderance of male patients who sustain these injuries [1]. Our observational group was younger (11.6 years old) than our experimental group (13.8 years old). Despite this, the BMI of the observational group was almost three points higher in our observational group. This could potentially confound our weight-bearing and immobilization data, however we were unable to find any evidence in the literature that increased BMI in this age group would contribute to delayed weight bearing [11,12]. Further studies including a larger sample size with less demographic variation may aid in the recognition of any BMI-associated complications.

Additionally, given our retrospective study design, we are unable to randomize our patients to groups and thus we could potentially have a demographically different subset of patients between groups. This could be demonstrated in our difference in ages between groups (11.6 vs 13.8). Our retrospective review suffers from the same disadvantages as similar studies relying on the electronic medical record to garner information. It also lacks patient-reported outcomes which would be pivotal in determining differences between subjective outcomes. We did not evaluate certain surgical characteristics for comparison such as operative time, blood loss, or tourniquet time. Significant variability occurred in the follow-up duration between patients, with a mean follow-up period of 43.6 and 108.1 weeks, so we were not able to effectively compare the ultimate healing rates between groups, nor follow them to skeletal maturity. We did however note that all patients in the study achieved satisfactory union by final follow-up. A prospective, randomized controlled trial between institutions that follows patients to skeletal maturity would potentially provide enough power for an additional statistically significant study to further contribute to the literature of tibial shaft fracture treatment in adolescents.

## Conclusions

Based on our small pilot study, RIMNs in adolescents should be considered as a potential treatment option when a physeal-sparing distal start point is utilized. Additionally, short-term follow-up has shown RIMNs in adolescents as a safe option and patients who underwent the RIMN procedure required fewer reoperations compared with the observational group. Overall, fracture healing was similar across the two groups. The benefits of RIMN include early immobilization and an improved weight-bearing profile.
